# Anti-fibrotic Effect of Oral Versus Intraperitoneal Administration of Gold Nanoparticles in Hepatic *Schistosoma mansoni-*Infected Mice

**DOI:** 10.1007/s11686-023-00730-w

**Published:** 2023-11-15

**Authors:** Shahira Abdelaziz Ali Ahmed, Samer Eid Mohamed Gad, Omima Mohamed Eida, Laila Mohamed Makhlouf

**Affiliations:** Department of Parasitology, Faculty of Medicine, Suez Canal University, Ismailia, 41522 Egypt

**Keywords:** *Schistosoma mansoni*, Liver, Gold nanoparticles, Intraperitoneal administration, Oral administration, Histopathology

## Abstract

**Background:**

Schistosomiasis significantly impacts public health, as it causes severe morbidity. Infections caused by *Schistosoma mansoni* (*S. mansoni*) can be treated with gold nanoparticles (AuNPs). This study aims to determine the most effective route of AuNPs administration and the magnitude of its anti-fibrotic effect.

**Methods:**

In the five groups' *in vivo* assay design, AuNPs were administered intraperitoneally (1 mg/kg) and orally (1 mg/100 g) to *S. mansoni*-infected mice. Biochemical parameters (serum levels of albumin and liver enzymes alanine aminotransferase (ALT), and aspartate aminotransferase (AST) were measured. The histological changes of the liver in distinct groups were evaluated using Hematoxylin and Eosin, Masson's trichrome, and immunohistochemical stains.

**Results:**

Infection with *S. mansoni* was associated with substantial changes in the histological architecture of liver tissue and abnormal levels of hepatic function tests (albumin, AST, and ALT). *Schistosoma* infected hepatocytes exhibited an abnormal microscopic morphology, granuloma formation and aggressive fibrosis. AuNPs restored the liver histological architecture with a highly significant anti-fibrotic effect and significantly corrected hepatic function test levels. Intraperitoneal administration of AuNPs resulted in the most significant anti-fibrotic effect against hepatic *S. mansoni* infection as observed in all histological sections with Masson's trichrome being the best stain to represent this fact.

**Conclusion:**

For treating *S. mansoni*-induced chronic liver fibrosis, intraperitoneal administration of AuNPs is a successful and effective route of administration that can be recommended.

## Introduction

*Schistosomiasis* is a parasitic disease caused by blood-dwelling trematode worms of *Schistosoma* [1]. There are six major human-infecting species, with *Schistosoma mansoni* (*S. mansoni*), *S. haematobium*, and *S. japonicum* accounting for the preponderance of human disease burdens [2].

According to the World Health Organization's most recent estimates, 78 nations and territories are still at risk for schistosomiasis, and around 240 million individuals needed preventative chemotherapy in 2020, with 91% of these patients residing in Africa [3]. In Africa, *S. mansoni* has the highest estimated prevalence range (HEPR) of over 50%, making it one of the continent's most common helminths [4].

It is estimated that 11,792 people die from schistosomiasis each year worldwide. These numbers, however, need to be considered [5]. Over 200,000 people in Sub-Saharan Africa die yearly due to schistosomiasis [5, 6]. The *S. mansoni* HEPR in Egypt was between 1 and 10% [4]. To limit transmission, the Egyptian Ministry of Health and Population (MoHP) improved the treatment technique far beyond what the World Health Organization (WHO) recommended. But significant transmission remained in a few loci [7].

Parasite eggs can be retained in the liver, gut, or reproductive organs, triggering an inflammatory response that leads to schistosomiasis [8]. Because the parasite ova affect the histological architecture of the liver, kidneys, and spleen, *Schistosoma* infection leads to oxidative stress in these organs [9]. Schistosomiasis symptoms can include abdominal pain, diarrhea, and bloody feces, depending on the body's response to the eggs. In chronic infections, the embolization of *Schistosoma* eggs into the liver and portal system results in chronic inflammation, tissue injury, and progressive tissue fibrosis. Advanced disease is distinguished by extensive periportal fibrosis, portal hypertension, varices, upper gastrointestinal bleeding, splenomegaly, and ascites [10–12].

*S. mansoni*-hepatic schistosomiasis results from the host's granulomatous cell-mediated immune response to the soluble egg antigen. Although granuloma formation is advantageous for the host because it inhibits the hepatotoxic effects of antigens released from parasite eggs, this process may result in periportal fibrosis due to the excessive accumulation of collagen and extracellular matrix proteins [13].

Praziquantel (PZQ) tablets are used to treat and manage schistosomiasis [14]. However, it only reaches around thirteen percent of its intended audience, and it is not suggested for kids under six because the tablets are so big and bitter [15]. Patients with severe infections have less success with PZQ, and it is ineffective at preventing reinfection [16]. Resistance to PZQ by *Schistosoma* has been documented due to reduced cure rates in newly exposed or heavily infected populations [17]. Pathological squeals in the gut, liver, brain, and kidney due to schistosomiasis cannot be reversed by PZQ [18].

Numerous nanoparticles (NPs) were utilized in the treatment of schistosomiasis [19–22], either as anti-parasitic agents or as protective agents against organ dysfunction induced by *S. mansoni*, such as hepatic dysfunction [23], renal dysfunction [24], neuro-dysfunction [25], or intestinal dysfunction [26]. Gold nanoparticles (AuNPs) are among the most significant in the field because of their many desirable properties, including inertness, biocompatibility, and especially low toxicity [27]. It has been extensively used in various nanomedicine fields for diagnostic and therapeutic purposes [22]. In a previous study [28], AuNPs were used to deliver nuclear-targeted pharmaceuticals into biological cells. In schistosomiasis, AuNPs were utilized to restore the expression of kidney-damaged genes [24] and reduce the total worm and egg burden in mice infected with *Schistosoma* [23].

Several research, including those using AuNPs administered intraperitoneally [23] and orally [29], have focused on determining if AuNPs can improve *Schistosoma*-liver fibrosis *in vivo*. However, research has been lacking on the optimal method of administering AuNPs to treat liver fibrosis. The current research aimed to determine the best way to help administer AuNPs for their hepatic anti-fibrotic effects and to determine to which extent AuNPs can ameliorate the liver fibrosis that *S. mansoni* infection *in vivo* causes.

## Methodology

### Animals’ Infection with *S. mansoni* Cercariae

At the Schistosome Biological Supply Center (SBSC), Theodor Bilharz Research Institute (TBRI) in Giza, Egypt, 35 male Swiss Albino mice aged nine weeks and weighing an average of 20 ± 2 g were maintained under specified pathogen-free conditions and fed a standard diet. Diet and water were provided ad libitum. Cercariae were acquired in suspension form after counting from TBRI [30]. Forty ± 10 *S. mansoni* cercariae were subcutaneously administered to mice [31–34]. The infection was confirmed by the presence of *S. mansoni* eggs in the feces of mice 42 days after initial infection [35].

### Preparation and Characterization of the Drugs

#### Praziquantel

Orally, an aqueous suspension of 600 mg PZQ tablets (purchased from Praziquantel-Sedico Pharmaceutical co. on the 6th of October City, Egypt) was administered in 2% Cremophor El. Cremophor El is a synthetic, non-ionic surfactant capable of stabilizing emulsions of non-polar substances in aqueous systems. Eight weeks after infection, a 0.25 mg/day dose of PZQ was administered for three days [36]. Before issuing the drug orally to mice using stainless steel oral gavages, it was freshly prepared [37].

#### Gold Nanoparticles (AuNPs)

Citrate-capped gold nanoparticles (NT-AuNPs) were purchased from NanoTech Egypt for Photo-Electronics, 6th of October City, Egypt. Using a chemical reduction method, AuNPs have been produced. Chloroauric acid (HAuCl_4_) has been used as a precursor for Au^3+^ ions, while sodium citrate has been used as a mild reducing and stabilizing agent. As Au^3+^ ions were converted into AuNPs, the color of the solution gradually changed to a subtle pink hue [38].

UV-V absorption spectra were obtained using an ocean optics uSb2000 + VIS–NIR fiber optics spectrophotometer to determine optical properties. The size and shape of AuNPs were determined by electron transmission microscopy [23].

### Experimental Design and *In Vivo* Drug Interventions

After infection, mice were randomly divided into five experimental groups (7 mice per group),

- G1, uninfected control (UC).
- G2, infected non-treated (InT) with phosphate-buffered saline (PBS) administration.
- G3, infected and treated with PZQ (IT-PZQ).
- G4, infected and treated orally with AuNPs (IT-AuNPs-Oral).
- G5, infected and treated intraperitoneal with AuNPs (IT-AuNPs-Peritoneal).

The *in vivo* assay is outlined in detail (Table 1). To establish chronic schistosomiasis, all infected mice were maintained for eight weeks after infection [36]. G4 and G5 were treated previously with PZQ [37]. The absence of *S. mansoni* ova in the feces of mice confirmed that the infection had been eradicated before the administration of AuNPs [39]. Doses were outlined in detail (Table 1) [23, 29, 40]. All mice were euthanized four weeks after the administration of AuNPs [23, 29].

**Table 1:** Design and outcome of the *in vivo* assay

Groups	Time of infection/treatment	Route of inoculation	Dose/Inoculate	Inoculate timeline	No. of infected mice	No. of living mice/ No. of total mice*	No. of cured mice from *S. mansoni* infection (%)
G1: UC	No inoculums	Intra-dermal	300 µL PBS per mice	One time	0/7	7/7	Uninfected
G2: InT	Day 1	Intra-dermal	40 ± 10 *S. mansoni* cercariae	One time	7/7	5/7	0/7 (0%)
G3: IT-PZQ	8 weeks *p.i*	Intragastric	250 mg/kg PZQ/animal	Once daily for 3 days	7/7	6/7	6/7 (85.7%)
G4: IT-AuNPs-Oral	9 weeks *p.i*^1^	Oral	1 mg/100g BW AuNPs /animal	Once daily for 4 weeks	7/7	6/7	6/7 (85.7%)
G5: IT-AuNPs-Peritoneal	9 weeks *p.i*^1^	Intraperitoneal	1 mg/kg AuNPs /animal	Two doses with 48 h interval	7/7	7/7	7/7 (100%)

G: Group; *p.i:* Post-infection; UC: Uninfected control; IT-PZQ: Infected and treated with praziquantel; AuNPs: Gold nanoparticles; IT-AuNPs-Oral: Infected and treated orally with gold nanoparticles; IT-AuNPs-Peritoneal: Infected and treated intraperitoneally with gold nanoparticles; PBS: Phosphate buffered saline; BW: Body weight; *S. mansoni*: *Schistosoma mansoni*

*The number of mice that perished did not affect the statistical equality of the groups, since each group had two mice drop out

^1^Mice were previously treated with PZQ

### Evaluation of Parameters Pertaining to Fibrosis

Different mice groups were assessed for liver fibrosis using fibrosis-related parameters, including liver function tests, hepatic histopathology, and morphometric measurements.

#### Measurement of Biochemical Parameters

Blood was collected in clean, dry test containers using a heparinized capillary tube by puncturing the retro-orbital plexus. Within eight hours of collection, samples were processed at ambient temperature. The tubes were allowed to stand for 30 min to clot at room temperature and then centrifuged (3,000 rpm for 15 min, 4 ± 2 °C). The serum was collected and used immediately for the determination of serum alanine aminotransferase (ALT), aspartate aminotransferase (AST), using colorimetric BioProm kits (Got/ALTL, and Got/ASTL-500 test reagents for Roche, Cobas, 6000, Germany) [41] and albumin using also BioProm kit (Got/ALB2-500 test reagents for Roche, Cobas, 6000, Germany) [42]. Units per liter (U/L) represent the results [43]. The serum levels of liver enzymes (ALT and AST) and albumin of all animal groups were measured, four weeks post-treatment (time of euthanization after treatment).

#### Histopathological Investigations

##### Hematoxylin and Eosin (H&E) Staining

Liver tissue samples of all groups were immediately fixed after animal dissection in 10% formalin, and Bouin’s fixatives, dehydrated and processed for paraffin sectioning [44]. The blocks were sectioned to a thickness of 4–6 µm [45]. For histopathological examination, sections were stained with H&E. H&E stain is the most commonly used medical diagnostic stain and the gold standard for hepatic histology diagnosis [46, 47]. Stained sections were examined and photographed using an Olympus light microscope [48].

###### Measurement of Liver Granuloma (Size and Diameter) [49]

Hepatic granulomas were calculated in five microscopic fields in serial tissue sections [50, 51]. The vertical and horizontal diameters of the hepatic granuloma containing a visible *S. mansoni* egg or egg fragment were measured using an ocular micrometer lens attached to a light microscope. The granuloma’s diameter was calculated as the mean of its vertical and horizontal diameters. For each mice group, the mean granuloma diameter (MGD) was calculated [52, 53]. Lesion counts between 50 and 100 µm were considered. The percentage of MGD suppression in the treated group was then determined [54].

###### Histological–Noninvasive Scoring of Liver Fibrosis

Histological grading was utilized to evaluate inflammatory changes in the morphological architecture of the liver as a result of *Schistosoma* infection. The absence, presence, and severity of fragmentary necrosis, confluent necrosis, apoptosis, focal inflammation, portal inflammation, fibrosis, and cirrhosis were evaluated to determine the histological scoring scores [55]. The highest potential grade is 18 points. The histological activity index was determined as follows: (1–3): minimal liver inflammation; (4–8): mild liver inflammation; (9–12): moderate liver inflammation; (13–18): severe liver inflammation. The scores of other inflammatory processes were (−: absent; +: mild; ++: moderate; and +++: severe).

##### Masson’s Trichrome Stain

Masson’s trichrome was used as another confirmatory stain to demonstrate the amount and pattern of collagen formation in the granuloma, which reflects the presence and distribution of reactive fibrosis due to hepatic injury [51, 56]. The stain was conducted in accordance with a previously mentioned protocol [44]. The area percentage of collagen content was determined in five randomly selected fields/sections for each mouse in each group at × 400 and × 1000 magnification. Using suitable image analysis software (Image Pro Plus, Photoshop), the area percentage of collagen was determined by isolating the blue color. The data were subsequently exported directly into Microsoft Excel, and a percentage was calculated [51].

#### Histochemical Investigations

Hepatic stellate cells (HSCs) are activated in fibroblasts and expressed the antibody alpha-smooth muscle actin (α-SMA) during liver fibrosis. Within and between granulomas, α-SMA-positive cells appeared as oval cells with brownish cytoplasm, signifying fibroblast proliferation and fibrosis [57]. Using a semiquantitative method [58, 59], the percentage area (%) of immunostained cells was determined by evaluating immunostained regions. Five random fields were selected from the liver section of each mouse to calculate the percentage of immunostained cell area (%) [57].

### Statistical Analysis

The data were presented as the mean, ± standard deviation (SD). One-way analysis of variance (ANOVA) was utilized to determine group differences. The symbol “*” indicates a significant difference (*p*-value < 0.05).

## Results

### Design and Outcome of the *In Vivo* Assay

The details and outcomes of the *in vivo* experiment are presented in Table 1. The treatment of *Schistosoma*-liver fibrosis was found to be most effective with the intraperitoneal administration of AuNPs resulting in complete cure of all infected mice. The efficacy of PZQ and AuNPs-Oral in the treatment of *Schistosoma*-liver fibrosis was shown to be comparable in a cure rate of 85%. The UC demonstrated being negative throughout the experiment (Table 1).

### Assessment of the Hepatic Function Parameters in the *In Vivo* Assay

**Fig. 1:**
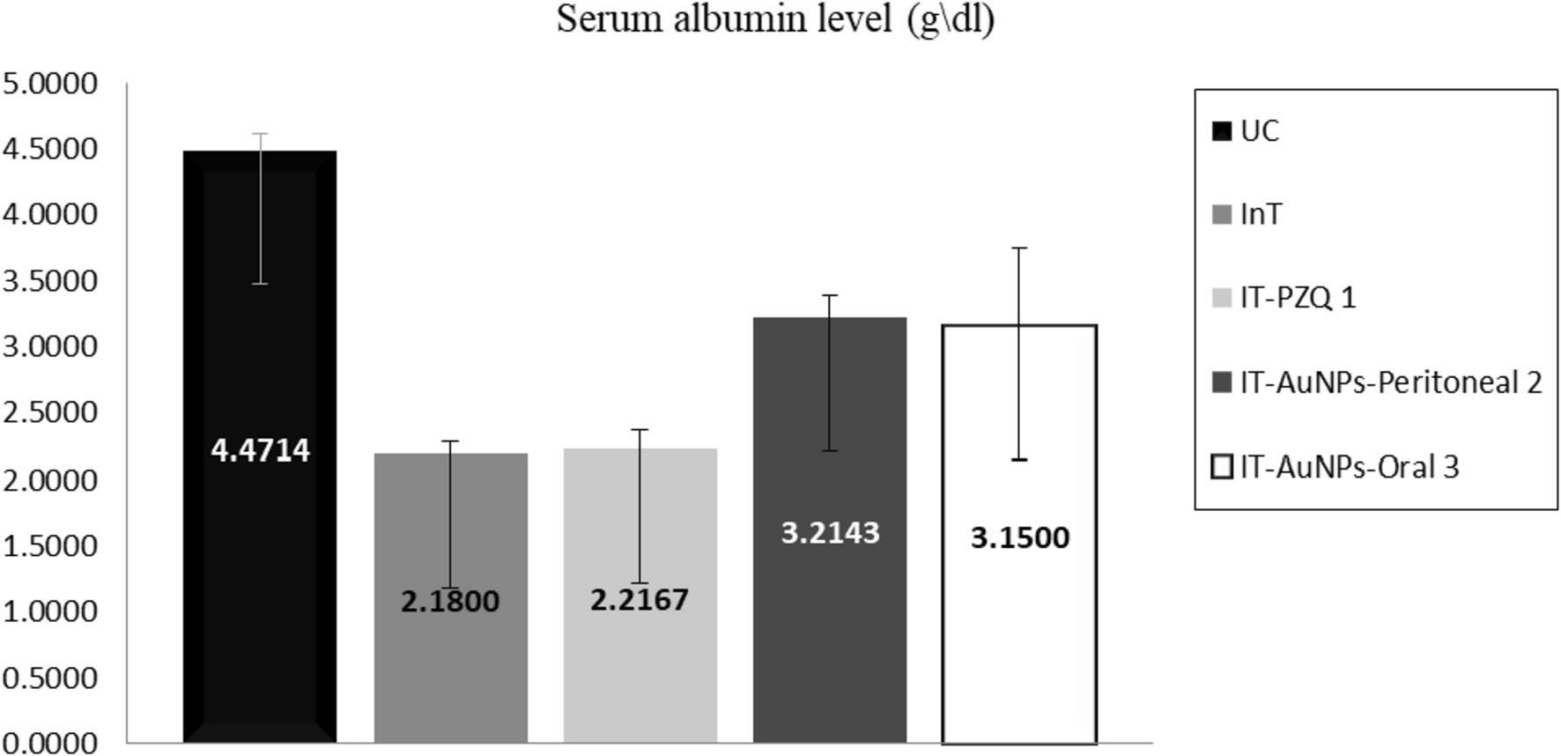
Serum albumin levels in the *in vivo* assay. ^1^Non-significant differences from IT-PZQ in comparison to InT group; ^2^Significant differences from IT-AuNPs-Peritoneal in comparison to InT group; ^3^Significant differences from IT-AuNPs-Oral in comparison to InT group

The liver function was evaluated by measuring albumin and liver enzyme (ALT and AST) levels in the mice’s serum. *S. mansoni* infection significantly decreased serum albumin levels compared to UC. The peritoneal and oral administration of AuNPs significantly increased (*p* =  < 0.05) serum albumin level to a level close to normal UC. However, intragastric administration of PZQ had almost no effect on the decreased level of serum albumin (Fig. 1).

*S. mansoni* infection (InT group) was associated with a significant increase in serum AST and ALT levels compared to the UC group (*p* = 0.000). In contrast, serum AST and ALT levels decreased significantly in the IT-AuNPs-Peritoneal group compared to the InT group. Whereas PZQ and AuNPs-Oral exhibited non-significant reductions in serum AST and ALT levels when compared to InT (Tables 2 and 3).

**Table 2:** Serum AST levels in the *in vivo* assay

Animal group	AST (U/L)	Reduction %	*p*-value
UC	25 ± 3.82	–	0.000^1^
InT	86 ± 21.31	**–**	
IT-PZQ^2^	74.16 ± 19.6	13.76%	
IT-AuNPs-Oral^3^	48.16 ± 8.26	44%	
IT-AuNPs-Peritoneal^4^	25.85 ± 1.95	69.94%	

^1^*p* value is statistically significant at<0.05; One-way ANOVA was the used test for the statistical analysis

^2^Non-significant differences from IT-PZQ in comparison to InT group

^3^Non-significant differences from IT-AuNPs-Oral in comparison to InT group

^4^Significant differences from IT-AuNPs-Peritoneal in comparison to InT group

**Table 3:** Serum ALT levels in the *in vivo* assay

Animal group	ALT (U/L)	Reduction %	*p*-value
UC	22.57 ± 0.97	**–**	0.0000^1^
InT	61.6 ± 10.45	**–**	
IT-PZQ^2^	58.5 ± 11. 5	5.03%	
IT-AuNPs-Oral^3^	51 ± 10.58	17.2%	
IT-AuNPs-Peritoneal^4^	35 ± 11.69	43.18%	

^1^*p* value is statistically significant at<0.05; One-way ANOVA was the used test for the statistical analysis

^2^Non-significant differences from IT-PZQ in comparison to InT group

^3^Non-significant differences from IT-AuNPs-Oral in comparison to InT group

^4^Significant differences from IT- AuNPs-Peritoneal in comparison to InT group

### Effect of AuNPs and PZQ on the Hepatic Histopathology and Morphometric Measurements

#### *S. mansoni*-Liver Granuloma Count

IT-AuNPs-Peritoneal and IT-AuNPs-Oral groups exhibited a statistically significant reduction in liver granuloma count compared to the InT group. The IT-AuNPs-Peritoneal group exhibited the lowest number of granulomas compared to the other groups, while the administration of PZQ resulted in the least reduction of *S. mansoni* granulomas count (Table 4).

**Table 4:** The number of granulomas count per five fields

Animal group	No. of granuloma per 5 field	*p*-value
InT	22.00 ± 1.58	0.000^1^
IT-PZQ^2^	17.83 ± 2.3	
IT-AuNPs-Oral^3^	11.5 ± 3.8	
IT-AuNPs-Peritoneal^4^	9.57 ± 1.5	

^1^*p* value is statistically significant at <0.05. One-way ANOVA was the used test for statistical analysis

^2^Non-significant differences from IT-PZQ compared to InT group

^3^Significant differences from IT-AuNPs-Oral compared to InT group

^4^Significant differences from IT- AuNPs-Peritoneal compared to Int group

#### Size / Diameter of *Schistosoma *Granulomas

The intraperitoneal administration of AuNPs has demonstrated a significant reduction in granuloma size (65.5% compared to the InT group) when compared to alternative treated groups. Although PZQ therapy has demonstrated a reduction in the size of granulomas associated with *Schistosoma* infection, it is essential to note that it is regarded as the least effective therapeutic option for reducing size of *Schistosoma* granulomas (Table 5).

**Table 5:** Diameter of hepatic granuloma in the *in vivo* assay

Animal group	Granuloma diameter (µm)	Reduction %	*p*-value
InT	306.4 ± 11.6	–	0.000^1^
IT-PZQ^2^	270 ± 14.14	11.8%	
IT-AuNPs-Oral^3^	190 ± 14.14	37.98%	
IT-AuNPs-Peritoneal^4^	105.7 ± 13.04	65.5%	

^1^*p* value is statistically significant at <0.05. One-way ANOVA was the test used for statistical analysis

^2^Non-significant differences from IT-PZQ compared to the InT group

^3^Significant differences from IT-AuNPs-Oral compared to the InT group

^4^Significant differences from IT-AuNPs-Peritoneal compared to the InT group

#### AuNPs-Alterations to the Histo-morphological Structure of Hepatic Schistosomiasis

##### Histological Grading of Fibrotic Changes

Based on the histological classification of Ishak *et al.* [55], the intraperitoneal administration of AuNPs led to a notable amelioration of the inflammatory processes caused by *Schistosoma* in the liver of the experimental group. This intervention resulted in the regression of the inflammatory process to a low level and returned it to a state of minimal inflammation. Closely, oral administration of AuNPs resulted in a mild to moderate reversal of the liver's *Schistosoma*-induced inflammatory processes (Table 6). PZQ has reduced inflammation; however, the effect was very marginal, maintaining a moderate level of inflammation.

**Table 6:** Histological grading and staging of *Schistosoma*-liver inflammation and fibrosis in the *in vivo* assay

Animal group	Histological activity index^*^	Necrosis / apoptosis	Dilated sinusoids	Infiltration of lymphocytes	Kupffer cells hyperplasia
UC	0	−	−	−	−
InT	15–18	** +++**	** +++**	** +++ **	** + ++**
IT-PZQ	9–12	** ++**	**++**	**++**	**++**
IT-AuNPs-Oral	5–10	** + **	** + **	**++**	** ++**
IT-AuNPs-Peritoneal	4–8	**+**	**+**	** +**	**+**

*According to Ishak *et al.* [55] the score details of the histological activity index were indicated. (1–3): minimal liver inflammation; (4–8): mild liver inflammation; (9–12): moderate liver inflammation; (13–18): severe liver inflammation. The scores of other inflammatory processes were (−: absent; +: mild; ++: moderate; and +++: severe)

##### H&E Stain-Illustrated Morphological Alterations in the *In Vivo* Assay

**Fig. 2:**
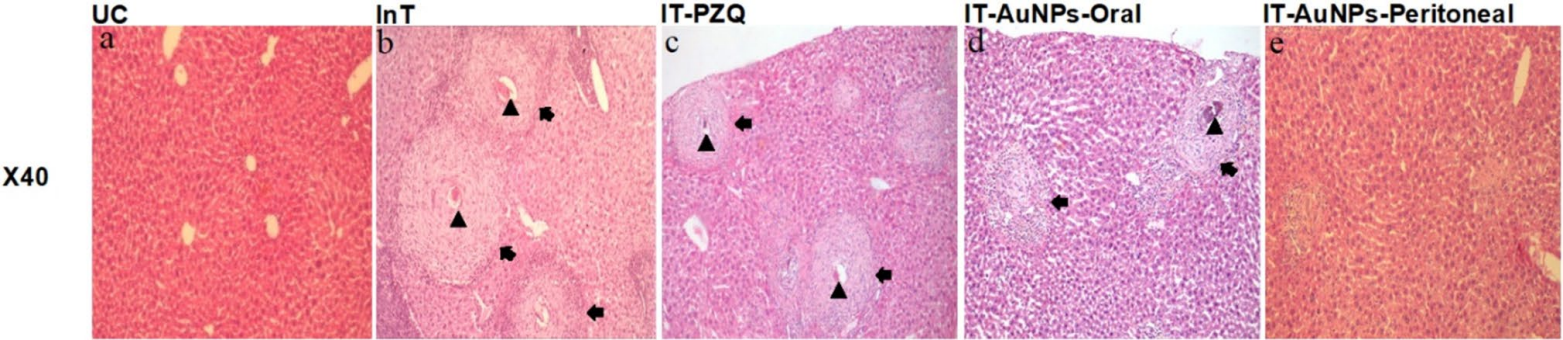
H&E stain-illustrated morphological alterations. The dry lens (x40) was used to examine morphological changes. The arrowhead indicates the trapped *Schistosoma* egg, and the arrow indicates the hepatic *Schistosoma* granuloma

Normal architecture was observed in the UC group, which contained intact classical hepatic cells with plates of hepatocytes radiating from the central vein and separated by blood sinusoids, no *S. mansoni* ova, and no inflammatory cells. (Fig. 2a). While in the InT group, sever inflammatory process was observed in the form of trapped *S. mansoni* ova between hepatocytes (large number, clear spine), numerous large and abundant granulomatous lesions (fibrous) around trapped ova, narrow liver parenchyma in between granulomatous lesions and severe inflammatory response (inflammatory cellular infiltration, cytoplasmic vacuolation, degeneration of hepatocytes, dilated hepatic sinusoids, hyper-eosinophilic hepatocytes and more Kupffer cells) (Fig. 2b). A moderate inflammatory process was observed in the IT-PZQ with trapped *S. mansoni* ova between hepatocytes (fewer, mostly viable and some dead), fewer granulomatous lesions (fibro-cellular), fewer inflammatory infiltrate, and larger part of liver tissue in between granulomas (Fig. 2c). A mild to moderate inflammatory process was induced in the IT-AuNPs-Oral with few dead, hyalinized and/or calcified ova and a reduction in the number and size of granulomas (cellular) (Fig. 2d). For the IT-AuNPs-Peritoneal mild inflammatory process was observed, whereas *S. mansoni*-trapped ova disappeared, granulomas significantly decreased in number and size by being more cellular with almost no / less encircling inflammatory infiltrate and apparently normal hepatic lobular architecture was preserved (Fig. 2e) making the peritoneal administration of AuNPs the best cure for the *Schistosoma*-inflammatory processes in the liver of its group.

##### Masson’s Trichrome Stain-Illustrated Morphological Alterations in the *In Vivo* Assay

###### The Collagen Percentage Content

The percentage of collagen content in the *in vivo* test groups is displayed in Table 7. In the IT-AuNPs-Peritoneal group, there was a statistically significant reduction in fibrosis area, as measured by collagen percentage (7.2%), compared to the InT group (25.8%).

**Table 7:** The percentage of collagen content in the *in vivo* assay

Animal group	Area of collagen (%)	Reduction %	*p*-value
UC	1.3 ± 0.1	**–**	0.000^1^
InT	25.8 ± 0.9	**–**	
IT-PZQ^2^	16.8 ± 0.85	34.88%	
IT-AuNPs-Oral^3^	10.9 ± 0.84	57.7%	
IT-AuNPs-Peritoneal^4^	7.2 ± 0.78	72.09%	

^1^*p* value is statistically significant at <0.05. One-way ANOVA has been used for the statistical analysis

^2^Non-significant differences from IT-PZQ compared to InT group

^3^Significant differences from IT-AuNPs-Oral compared to InT group

^4^Significant differences from IT- AuNPs-Peritoneal compared to InT group

#### Masson's Trichrome Stain-Illustrated Images of Morphological Alterations

**Fig. 3:**
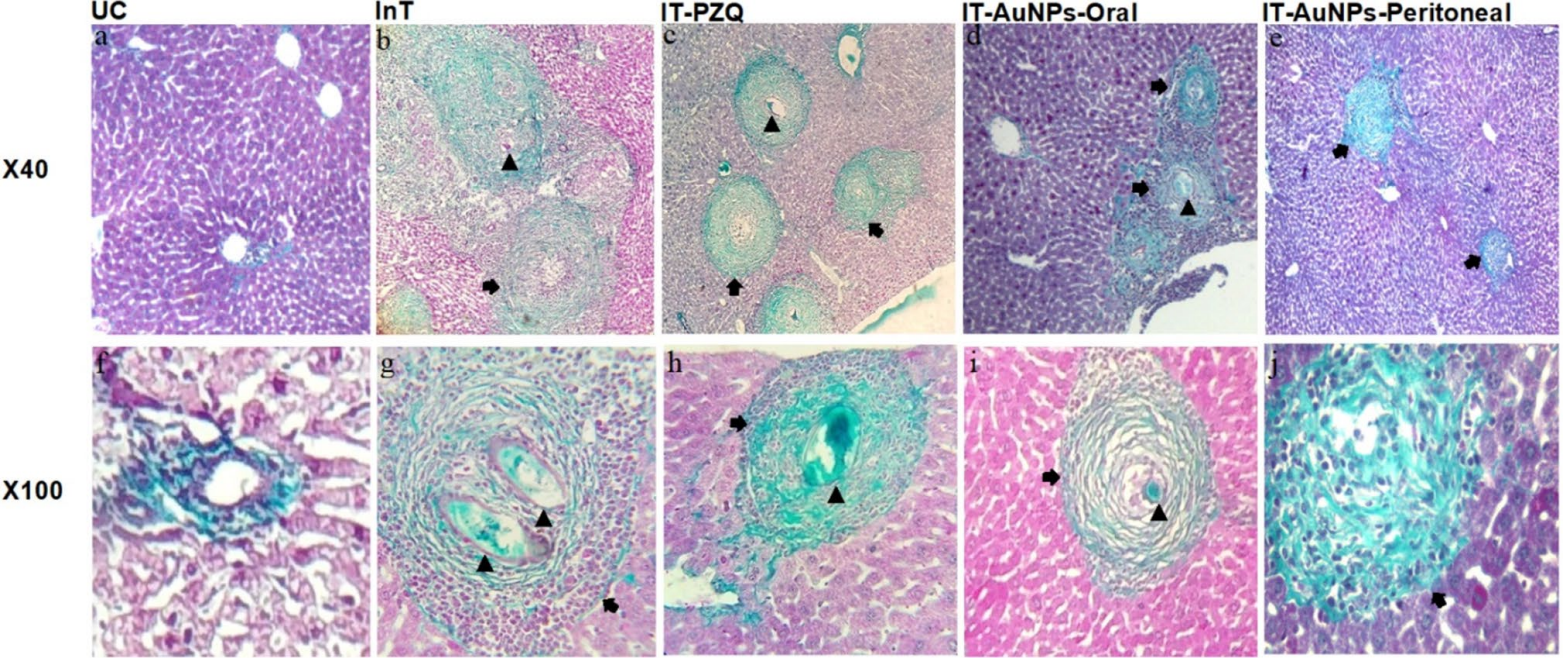
Masson's trichrome stain-illustrated morphological alterations. The dry and oil lens (x40 and x 100) were used to examine morphological changes. The arrowhead indicates the trapped *Schistosoma* egg, and the arrow indicates the hepatic *Schistosoma* granuloma collagen

In the UC group, normal liver architecture was observed with only a small number of collagen fibers typically found around central veins and portal regions. Figure 3a and f depict collagen fibers with a blue stain, a black nucleus and a red or purple background. In contrast, the InT group exhibited sever fibrosis with abundant collagen fibers arrayed in multiple directions in some granulomas and marked accumulation with concentric orientation of collagen fibers in others (Fig. 3b, g). A moderate fibrosis was observed in the IT-PZQ group, with a minor decrease in the amount of collagen fibers in the granulomas, and collagen fibers appeared as fragmented fibers and dispersed into a loose amorphous matrix (Fig. 3c, h). In the IT-AuNPs-Oral group, mild to moderate fibrosis was observed in the image of reduced fragmented collagen fibers, particularly in the center of granulomas (Fig. 3d, i). The peritoneal administration of AuNPs was the most effective treatment for the *Schistosoma*-inflammatory processes in the liver of its group, as evidenced by outstanding morphological regression of the disease with sparse collagen fibers and mild fibrosis (Fig. 3e, j).

Masson's trichrome stain provided transparent images for identifying *Schistosoma*-morphological alterations in comparison to H&E stain.

##### Immunohistochemical Stain-Illustrated Morphological Alterations in the *In Vivo* Assay

###### Percentage of α-SMA in the Studied Groups

The liver tissue of mice in the InT group exhibited a considerable increase in α-SMA deposition in portal areas and intrahepatic sinusoids. The expression of α-SMA has decreased significantly in the IT-AuNPs-Peritoneal group (12%) compared to the InT group (22%) (Table 8). The percentages of α-SMA in other groups are shown in Table 8.

**Table 8:** α-SMA percentage within the examined groups

Animal group	Area of α-SMA (%)	Reduction %	*p*-value
UC	1.4 ± 0.31	**–**	0.000^1^
InT	22 ± 1.58	**–**	
IT-PZQ^2^	18 ± 1.41	18.18%	
IT-AuNPs-Oral^3^	14.16 ± 2.8	35.63%	
IT-AuNPs-Peritoneal^4^	12 ± 2.6	45.45%	

^1^*p* value is statistically significant at <0.05. One-way ANOVA has been used for the statistical analysis

^2^Significant differences from IT-PZQ compared to InT group

^3^Significant differences from IT-AuNPs-Oral compared to InT group

^4^Significant differences from IT-AuNPs-Peritoneal compared to InT group

#### Immunohistochemical Stain-Illustrated Images of Morphological Alterations

**Fig. 4:**
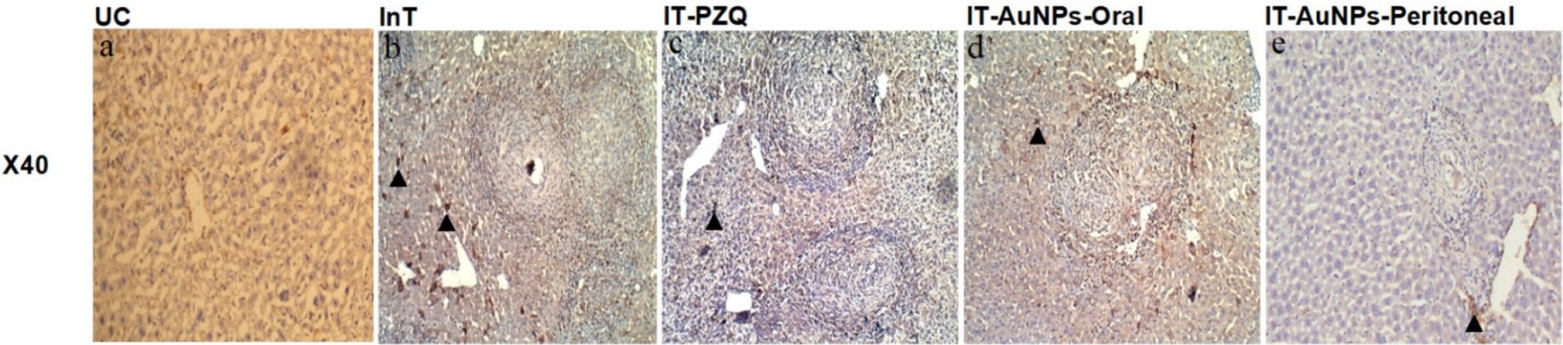
Masson's trichrome stain-illustrated morphological alterations. The dry lens (x40) was used to examine morphological changes. The arrowhead indicates brownish immune-stained regions

According to Fig. 4, the UC group’s liver architecture was normal with α-SMA-positive cells only in tunica media of central veins and fusiform cells surrounding bile ducts (Fig. 4a). In contrast, the InT group displayed extensive fibroblastic proliferation with numerous brownish regions of immunostained cells within and between granulomas (Fig. 4b). The IT-PZQ group demonstrated moderate fibroblastic proliferation with mild decrease in brownish immunostained regions (Fig. 4c). In the IT- AuNPs-Oral group, the immunostained regions were moderately reduced (Fig. 4d). The peritoneal administration of AuNPs was the most successful treatment indicating mild fibroblastic proliferation and obvious marked decrease in area of immunostained regions (Fig. 4d).

## Discussion

AuNPs have recently begun to be utilized extensively in numerous nanomedicine sectors for diagnostic and therapeutic applications [22–24, 27, 28, 60]. The current study aimed to determine the anti-fibrotic effect of AuNPs as well as the most effective route of AuNPs administration for the mitigation of *S. mansoni*-induced liver fibrosis *in vivo*.

Current research indicates that regardless of the route of administration, AuNPs are generally very effective agents in ameliorating *S. mansoni*-induced liver fibrosis. Another study, such as Dkhil *et al.*, indicated that AuNPs reduce schistosomiasis-induced oxidative stress and may treat hepatic dysfunction [23]. AuNPs also ameliorated carbon tetrachloride (CCl4)-induced liver fibrosis in rats, improving liver function and histopathology compared to silymarin-treated groups [29]. Like to Kabir *et al.*, silymarin-coated AuNPs improved CCl4-induced liver damage and cirrhosis [61]. In alcohol-methamphetamine-induced liver damage in rats, AuNPs also caused anti-inflammatory response, antioxidant stress, and anti-fibrosis [62].

The anti-fibrotic effect of AuNPs may be due to their ability to down-regulate the human hepatic stellate cells (HSC) and Kupffer cells. Kupffer cells recognize the nanoparticles and accumulate them in the liver as opsonins via interaction with its receptors [63, 64]. AuNPs migrate to the Kupffer cell nucleus where they negatively modulate cytokine release and modulate the pathways of phosphatidylinositol 3-kinase/AKT and mitogen-activated protein kinases that play an essential role in the occurrence and development of liver fibrosis by controlling the degradation of collagenous extracellular matrix and activation of HSC [62, 65].

AuNPs were used in two distinct routes (oral and intraperitoneal) in the present study. Both AuNPs’ ways effectively reduced *S. mansoni*-induced liver fibrosis *in vivo*, provided that the intraperitoneal route was the most effective. Such results were notably observed by substantially enhancing the functions of the liver and removing fibrotic lesions in the histopathological sections relative to the InT group and the standard PZQ treatment group.

Liver inflammation and necrosis were marked with serum albumin, AST, and ALT levels [66]. In the present study, they were utilized to assess hepatic function and liver tissue improvement. The InT group had severe schistosomiasis-liver dysfunction, with a severe drop in serum albumin and a considerable increase in serum AST and ALT. Other research found that schistosomal infection elevated plasma ALT and AST levels [67, 68]. Changes in serum albumin, AST and ALT levels can be clarified as *S. mansoni*-associated-hepatic dysfunction involves impaired protein synthesis and elevated circulating levels of liver enzymes [69, 70]. In the current study, intraperitoneal AuNPs improved liver function more than other groups (IT-AuNPs-Oral and IT-PZQ) by increasing serum albumin, decreasing AST, and increasing ALT. Other studies have indicated that intraperitoneal AuNPs are better than PZQ for ameliorating liver damage and enhancing its function [61, 62, 68].

The present work used H&E, Masson trichrome, and immunohistochemical stains to show histopathological alterations in liver sections of all groups. Because each stain displayed histopathological alterations differently, using multiple stains validated the findings. For example, H&E was used to stage liver fibrosis [46], Masson's trichrome stain delineates injury patterns by showing the presence and distribution of reactive fibrosis due to liver damage [56], and immunohistochemical staining was used to measure α-SMA percentage suggesting fibroblastic proliferation and fibrosis [56].

In the current study, *S. mansoni* infection caused significant inflammation with several giant granulomas in the InT group, as shown by H&E stain. Some granulomas have extensive fibrosis with collagen fibers structured in numerous directions, whereas others have concentric collagen fibers, as demonstrated by Masson's trichrome stain. Marked and active fibroblastic proliferation with brownish patches of immunostained cells within and between granulomas has been observed, as shown by immunohistochemical stain. Such inflammatory process has been similarly documented and detailed in several research studies [19, 71–76]. A broad range of liver damage had been identified in schistosomiasis, and liver fibrosis was a typical finding in advanced schistosomiasis mainly at the site of ongoing granulomatous reactions [77, 78]. The extensive inflammatory process in the InT group of the present study has returned to the intensity of infection, as mice were infected non-treated for eight weeks. Granulomas usually arise as a result due to ova deposition in the tissue. Therefore, their sizes and numbers are associated with the intensity of *S. mansoni*-chronic infection [79].

In the current study, the intraperitoneal administration of AuNPs significantly improved the histopathological findings of *S. mansoni*-infected mice (the inflammatory process became mild). There was a notable reduction in the number and extent of granulomas, and the lobular architecture of the liver appeared normal. Soft inflammatory processes were observed in the absence of *S. mansoni*-trapped ova. A marked decrease in the number and size of granulomas, and an apparently normal hepatic lobular architecture by H&E stain, mild fibrosis with scanty collagen fibers in small granulomas by Masson's trichrome stain, and mild fibroblastic proliferation and a marked decrease in immunostained area by immunohistochemical stain were observed. Other studies were consistent with our findings [23, 61, 80, 81]. IT-AuNPs-Peritoneal's profound impact on *S. mansoni*-associated liver fibrosis might return to the intraperitoneal route's advantages and the chemical properties of AuNPs. Intraperitoneal drug administration is both safe and efficacious for delivering large quantities of the drug. A drug that is absorbed from the peritoneal cavity by the portal system is subjected to hepatic first-pass elimination. The rapid uptake from the peritoneal cavity would result in a faster saturation of the AuNPs metabolizing enzymes. The intraperitoneal route permits hepatic processing before systemic circulation, and a small amount of intraperitoneal injectate can travel directly to the thoracic lymph [82–84].

The oral route of AuNPs in the present study proved to be effective in reducing *S. mansoni* hepatic damage; however, it was less effective than the intraperitoneal route (the inflammatory process has turned out to mild–moderate). In rats with CCl4-induced liver fibrosis, oral AuNPs improved histopathological findings [29]. Due to the ease of administration, continuous delivery and capacity for solid formulations with a long shelf life, the oral route is commonly accepted as an attractive and preferred method of administration [85, 86]. The oral route, however, may have adverse effects due to non-specific cellular uptake poor therapeutic effectiveness due to their instability during digestion and absorption [85]. The challenges of drug absorption/efficacy do not limit the barriers met in the gut, but they include the hepatic barriers after they enter the vessels under the intestinal epithelium [86, 87]. The oral administration of AuNPs was maintained for 28 consecutive days in the present study to demonstrate a satisfactory effect on *S. mansoni*-chronic liver fibrosis, which is very effortful and prolonged.

In the present study, PZQ, although it was regarded as the standard medication for treating *S. mansoni*-chronic fibrosis, showed a poor effect on hepatic histopathology. It caused a minimal decrease in the size and diameter of the granuloma. Moderate inflammatory processes were seen in the IT-PZQ group when stained with H&E. There was a slight decrease in the amount of collagen fibers. In contrast, fibers appeared fragmented and dispersed into a loose amorphous and abundant matrix with Masson's trichrome stain. Moderate fibroblast proliferation was also observed, with a mild decrease in brownish immunostained areas in the immunohistochemical stain. The poor effect of PZQ was also recorded in mice CCl4-driven liver fibrosis model, mice chronic liver fibrosis schistosomiasis model, and novel human cell-based co-culture; PZQ did not significantly reverse fibrosis [88]. In the current study, PZQ was administered as 250 mg/kg gavage once daily for three days, beginning eight weeks after infection. In contrast, Liang *et al.* demonstrated the anti-fibrotic effect of PZQ in *S. japonica*-infected murine models, albeit with a higher dose and over an extended period [36]. While PZQ was administered to alleviate hepatic fibrosis during the early chronic phase of infection (14 days post-infection) [89].

This disparity in dosage, duration, and treatment initiation may explain the inadequate anti-fibrotic effect of PZQ in the present study. The ability of PZQ to modulate the expression of several cytokines, but its inability to influence the expression of myofibroblast activation markers and associated matrix fibrosis activities such as α-SMA and collagen [88, 90] is another plausible explanation for PZQ's inability to effectively reverse the already-developed severe tissue fibrosis and digestion of deposited collagen.

## Conclusion

AuNPs ameliorated liver fibrosis in *S. mansoni*-infected mice with a pronounced anti-fibrotic effect. The intraperitoneal route of AuNPs is a successful and effective route that can be recommended for treating *S. mansoni*-induced chronic liver fibrosis. Significant liver regeneration is observed with this route, but additional research into dose variability, therapeutic mechanisms, and adverse effects would be advantageous. It is also a simple and cost-effective route of administration, as only two doses are necessary to attain drug efficacy, as opposed to 28 days of oral administration and the ineffectiveness of PZQ. It was determined that Masson's trichrome stain was the most effective stain for determining the phase of liver fibrosis, as it disclosed very specific liver granulomas, *Schistosoma* eggs, and collagen zones. Masson's trichrome stain could therefore be used as a differentiated stain to monitor the effects of various agents on liver fibrosis.

## Data Availability

All data generated or analyzed during this study are included in this published article [and its supplementary information files].
